# Plasma proteins and different onset subtype of COPD: Proteome-wide Mendelian randomization study and co-localization analyses

**DOI:** 10.1097/MD.0000000000042409

**Published:** 2025-05-09

**Authors:** Xu Chu, Zhifa Han, Baicun Li, Ting Yang

**Affiliations:** a National Center for Respiratory Medicine; State Key Laboratory of Respiratory Health and Multimorbidity; National Clinical Research Center for Respiratory Diseases; Institute of Respiratory Medicine, Chinese Academy of Medical Sciences; Department of Pulmonary and Critical Care Medicine, Center of Respiratory Medicine, China-Japan Friendship Hospital, Beijing, P.R. China; b Department of Pulmonary and Critical Care Medicine, The First Affiliated Hospital of Henan University of Science & Technology, Luoyang, P.R. China.

**Keywords:** chronic obstructive pulmonary disease, colocalization, genome-wide association studies, Mendelian randomization, plasma proteins

## Abstract

Several studies have reported a strong association between plasma proteins and chronic obstructive pulmonary disease (COPD). However, the directionality and causality of the association and whether proteins effected COPD remain unclear. Therefore, we used Proteome-wide Mendelian randomization (MR) study and co-localization analyses to estimate the casual relationship between them. Summary-level data of 2923 plasma protein levels were extracted from a large-scale protein quantitative trait loci study including 54,219 individuals by the UK Biobank Pharma Proteomics Project. The outcome data for COPD and its subtypes were sourced from the FinnGen study. MR analysis was conducted to estimate the associations between protein and COPD and its subtypes risk. Additionally, phenome-wide MR analysis, and candidate drug prediction were employed to identify potential causal circulating proteins and novel drug targets. STROBE MR guidelines are followed for the study. We assessed the effect of 1929 plasma proteins on COPD. We found that Seven proteins, 4 proteins, and 3 proteins were associated with overall COPD, early-onset COPD, and later-onset COPD risk, respectively. MHC class I polypeptide-related sequence B_A (MICB_MICA) and tyrosine-protein kinase receptor tie-1 (TIE-1) would increase 8% and 27% COPD risk (MICB_MICA: odds ratios [OR], 1.08; 95% CI, 1.05–1.10; *P*_FDR_ = 2.53 × 10^−5^; TIE-1: OR, 1.27; 95% CI, 1.13–1.43; *P*_FDR_ = .012). There was negative association of Septin-8 and Butyrophilin subfamily 1 member A1 (BTN1A1) with overall COPD risk (Septin-8: OR, 0.68; 95% CI, 0.57–0.79; *P*_FDR_ = 8.00 × 10^−4^ BTN1A1: OR, 0.82; 95% CI, 0.75–0.90; *P*_FDR_ = .010). There was a protective effect of BTN1A1 on early COPD incidence (OR, 0.72; 95% CI, 0.63–0.83; *P*_FDR_ = .002). However, there was no evidence indicating a shared causal variant between the other proteins and COPD and its subtypes in these regions (all posterior probability.H4 < .8). The study revealed the causal relationship between several plasma proteins and COPD and its subtypes, providing new theoretical support for understanding COPD.

## 1. Introduction

Chronic obstructive pulmonary disease (COPD) is a complex lung condition mainly characterized by persistent airflow limitation. It typically appears as chronic cough, sputum, shortness of breath, dyspnea, wheezing, and chest tightness caused by chronic bronchitis or emphysema.^[1]^ These symptoms significantly affect the quality of life of patients. Further progression of the disease can be complicated by pulmonary heart disease, respiratory failure or lung cancer, which is the leading cause of disability and death from COPD.^[2,3]^ COPD is currently the third leading cause of death worldwide, affecting approximately 600 million people, and the incidence will continue to increase with the trend of an aging population.^[4–6]^ Timely intervention before progression to irreversible disease effectively improves quality of life and reduces mortality. Therefore, exploring diagnostic and therapeutic targets for COPD is of great public health importance.

Many proteins leaked or actively secreted by cells are present in the blood, assuming critical roles in substance transport, tissue growth and repair, and signaling. Differences in the levels of these proteins under physiological or pathological conditions provide support for a deeper understanding of the mechanisms of disease and the exploration of new targets. It also contributes to the long-term prediction of drug effects, providing value in improving disease cure rates and reducing adverse drug events.^[7,8]^ Several studies have reported a strong association between plasma proteins and COPD. For example, a randomized controlled trial that included 101 patients with COPD demonstrated that the anti-IL-5 receptor blocker alpha showed some improvement in lung function in patients with high eosinophil counts.^[9]^ Another phase II clinical trial showed that CXCR2 antagonists reduced chemotaxis of neutrophils and attenuated Inflammation of the trachea in COPD.^[10]^ Several cross-sectional studies have also revealed high levels of TNF-α in the peripheral blood of COPD patients. In addition, relationships between COPD and mediators such as IL-1β, IL-8, IL-6, and IL-17 have been reported.^[11–14]^ However, the results of these studies are not all meaningful and may be due to small sample size, poor experimental design, and limited assay screening methods. Therefore, more comprehensive analyses are needed to explore the association between proteins and COPD.

Mendelian randomization (MR) leverages the randomness of genetic variation to overcome confounders and reverse causality bias in observational studies.^[15]^ It is widely used to predict the causal relationship between exposure and outcome. SNP identified by genome-wide association studies (GWAS) serve as instrumental variables (IVs) for causality analysis, providing robust evidence for identifying disease diagnostic markers and drug targets. It has been widely used to detect causal relationships between plasma proteins and various diseases.^[16–20]^ In this study, the causal effects of 2923 plasma proteins on COPD were explored using 2-sample MR analysis, and co-localization analysis verified the reliability of the results. In addition, the potential side effects of using these proteins as targets for drug therapy were assessed in this study in conjunction with Phenome-wide MR (Phe-MR) analysis.

## 2. Methods

### 2.1. Study design

**Figure 1.**
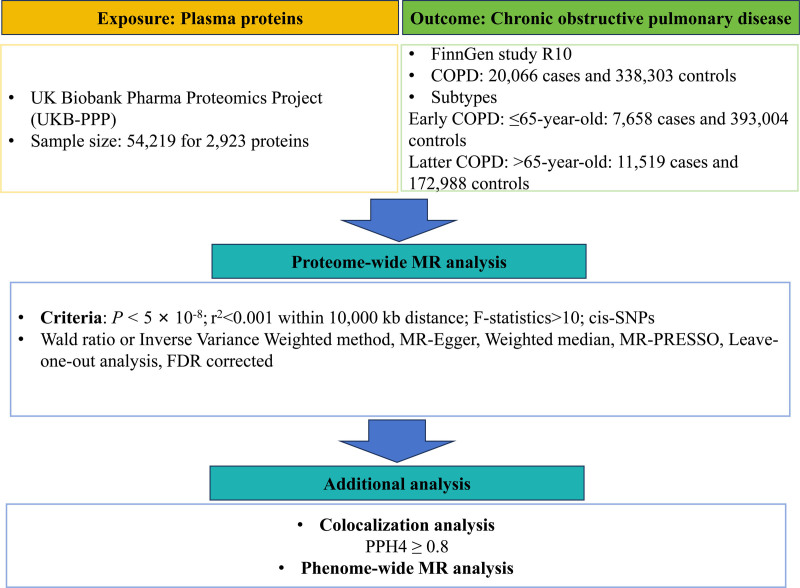
The comprehensive layout of the study. COPD = chronic obstructive pulmonary disease, FDR = false discovery rate, MR-PRESSO = MR pleiotropy residuals and Mendelian randomization pleiotropy residual sum and outlier, SNP = Single nucleotide polymorphisms, UKB-PPP = UK Biobank Pharma Proteomics Project.

Figure 1 illustrates the comprehensive layout of the study. We conducted 2-sample MR analyses to examine the association between plasma proteins and COPD using summary data from GWAS originating from studies. Co-localization analysis was employed to verify the causal associations between circulating proteins and cancer susceptibility. Finally, Phe-MR analysis was used to investigate potential side effects related to drug targets. Since our analysis exclusively relied on aggregated statistical information, additional ethical clearance was deemed unnecessary.

### 2.2. Exposure data for protein

The MR analysis in this study used data from the UK Biobank Pharma Proteomics Project (UKB-PPP),^[7]^ the largest global plasma proteome project GWAS (Table S1, Supplemental Digital Content, https://links.lww.com/MD/O872). The collaborative project between the UK Biobank and 13 biopharmaceutical companies aims to characterize the plasma proteome of 54,219 participants and conduct comprehensive localization of protein quantitative trait loci (pQTL) for 2923 proteins.^[7]^ It identifies 14,287 primary genetic associations, with 85% being novel, and observed independent secondary associations in 87% of cis and 30% of trans loci, expanding the genetic tool repository for further analysis.^[7]^

As cis-SNPs were considered to have a more direct and specific biological effect on the protein, we performed MR analyses using cis-SNPs as IVs.^[16,21]^ The following criteria were used to select instruments and proteins: Single nucleotide polymorphisms (SNPs) associated with any protein were selected (*P* < 5 × 10^−8^);^[22]^) To alleviate the influence of linkage disequilibrium (LD) on the independent SNPs, the threshold of LD parameter (*r*^2^) is .001 and a genetic distance is 10,000 kb;^[22]^ SNPs within 1Mb from the gene encoding the protein are defined as cis-SNPs. Finally, the strength of the IVs was measured using the *F*-statistic, and an *F*-statistic < 10 was considered a weak IV.^[16,21]^

### 2.3. Outcome data for COPD

The GWAS data for associations with COPD and its subtypes were sourced from FinnGen (https://www.finngen.fi/en), a large-scale genomic research project developed in Finland.^[23]^ Summary statistics for COPD (R10_J10_COPD cases: 20,066; controls: 338,303) in FinnGen, a consortium of Biobanks in Finland (Freeze 10. COPD was defined based on International Classification of Diseases codes retrieved from nationwide registries in Finland^[23]^ (Table S1, Supplemental Digital Content, https://links.lww.com/MD/O872). COPD subtypes in younger (7658 cases and 393,004 controls) and older (11,519 cases and 172,988 controls) FinnGen cohort were analyzed to evaluate the impact of leukocyte traits and age on COPD risk according to previous study^[23]^ (Table S1, Supplemental Digital Content, https://links.lww.com/MD/O872). The analyses in FinnGen were adjusted for age, sex, 10 principal components, and genotype batch using mixed‐model logistic regression [Scalable and Accurate Implementation of GEneralized mixed model (SAIGE)] by the FinnGen investigators.^[23]^ The 2 data sources (UK Biobank and FinnGen) representing European ancestry populations, showed comparable genetic associations.

### 2.4. Proteome-wide MR analysis

For the Phe-MR analysis, we used the inverse variance weighted (IVW) method with random effects as the primary approach to estimate the relationship between plasma protein and COPD.^[22]^ Each candidate SNP instrument utilized the Wald ratio to estimate its causal effect. For multiple SNPs, the simplest approach is to perform an IVW meta-analysis of each Wald ratio.^[24]^ In addition, we employed weighted median and MR-Egger methods to validate the accuracy of our results, which were presented as odds ratios (OR) and 95% confidence intervals and visualized using forest and heat plots. Sensitivity analysis played a crucial role in ensuring the robustness of MR analysis. Specifically, we assessed that the weighted median methods can be considered the causal effect estimate without bias when up to 50% IV invalid.^[25]^ The MR-Egger intercept was applied to detect horizontal pleiotropy and provided corrected estimates.^[26]^ MR pleiotropy residuals and MR pleiotropy residual sum and outlier (MR-PRESSO) were employed to identify potential outliers with horizontal pleiotropy and offered causal estimates after removing outliers.^[27]^ Leave-one-out analysis was used to determine whether individual SNPs influenced the MR results. The MR analysis was performed by using the R package of “TwoSampleMR” (https://github.com/MRCIEU/TwoSampleMR). The significant variants were the false discovery rate (FDR) for adjusted *P*-value below .05.

### 2.5. Co-localization analysis

Co-localization analysis determines if 2 share 2 traits share causal variants within a single region, testing 5 hypotheses: hypothesis 0 (H0): not associated with either trait; hypothesis 1 (H1): associated with trait 1, but not with trait 2; hypothesis 2 (H2): associated with trait 2, but not with trait 1; hypothesis 3 (H3): associated with both traits via independent SNPs; hypothesis 4 (H4): associated with both traits via shared SNP. A higher posterior probability (PP) of the H4 (PP.H4) supports significant MR results. In this study, co-localization analysis was conducted using the ‘coloc’ R package (https://github.com/chr1swallace/coloc).^[28]^ PP.H4 > .8 was defined as supporting evidence of co-localization analysis.

### 2.6. Phenome-wide MR analysis

To investigate the potential side effects of 4 drug targets, we used IVs from the UKB-PPP proteome as exposure (Table S2, Supplemental Digital Content, https://links.lww.com/MD/O873) and 2408 traits (diseases) from FinnGen as outcomes for Phe-MR analysis. From the FinnGen Public Documentation (https://finngen.gitbook.io/documentation/data-download) to download summary statistics of SNPs associated with the disease. The causal effects are considered statistically significant at FDR < .05. The report follows the statement “Strengthening the Reporting of Observational Studies in Epidemiology Using MR” (Table S7, Supplemental Digital Content, https://links.lww.com/MD/O874).^[29,30]^

## 3. Results

### 3.1. Mendelian randomization analysis

**Figure 2.**
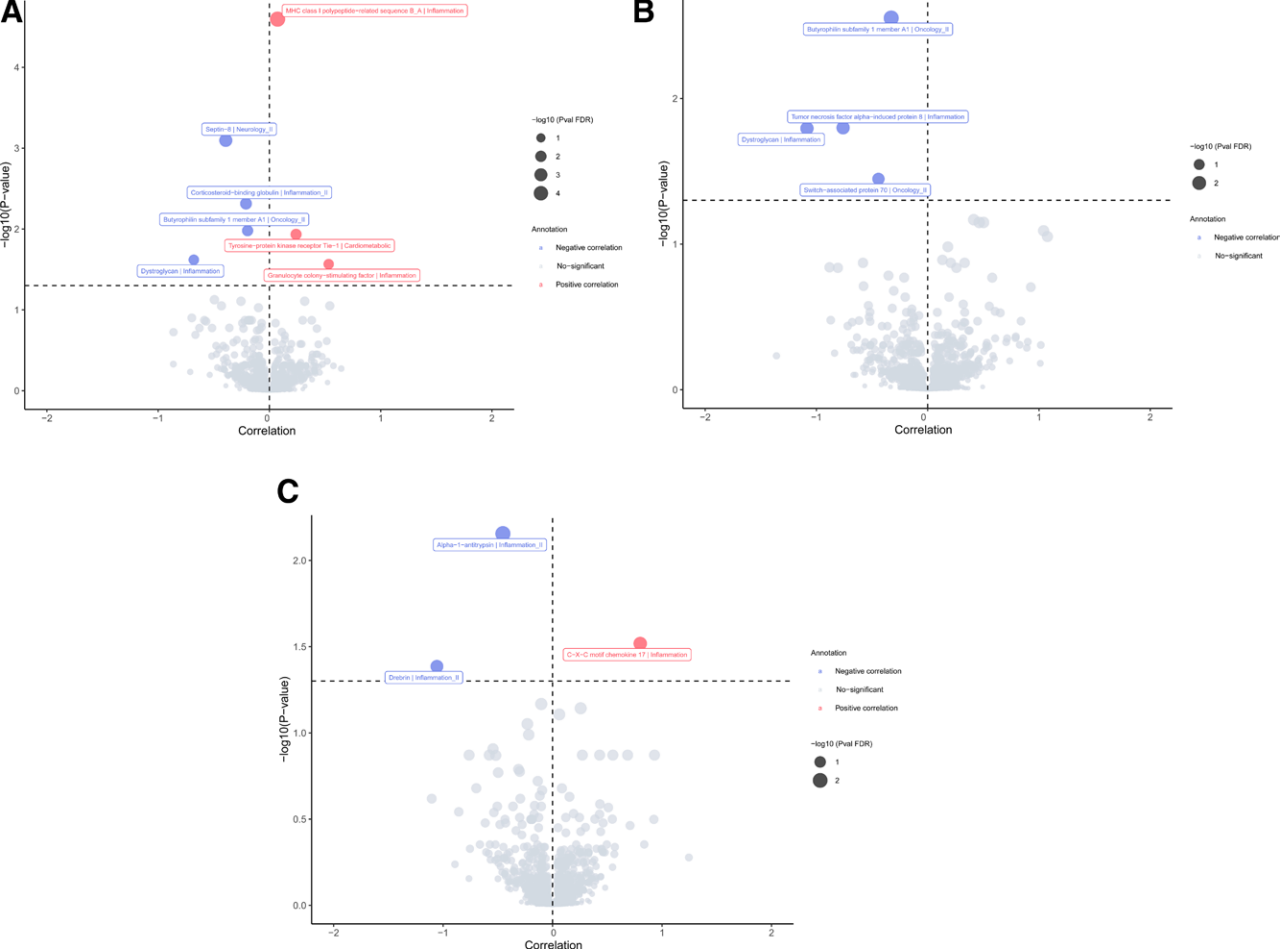
Proteins observed to be associated with COPD risk. Four proteins were negatively related to overall COPD risk, while 3 were positively associated with overall COPD risk (A). Four proteins would decrease the risk of early-onset COPD risk (B). Three proteins were associated with the later-onset COPD risk (C). COPD = chronic obstructive pulmonary disease, FDR = false discovery rate.

We selected 5647 SNPs as IVs for 1929 proteins, with F-statistic value > 10, suggesting that no weak instrument effect on the association between proteins with COPD and its subtypes (Table S2, Supplemental Digital Content, https://links.lww.com/MD/O873). Seven proteins, 4 proteins, and 3 proteins were observed to be associated with overall COPD, early-onset COPD, and later-onset COPD risk, respectively (Fig. 2). Four proteins were negatively related to overall COPD risk, while 3 were positively associated with overall COPD risk (Fig. 2A). For example, MHC class I polypeptide-related sequence B_A (MICB_MICA), tyrosine-protein kinase receptor tie-1 (TIE-1) and granulocyte colony-stimulating factor (G-CSF) would increase 8%, 27% and 70% COPD risk (MICB_MICA: OR, 1.08; 95% CI, 1.05–1.10; *P*_FDR_ = 2.53 × 10^−5^; TIE-1: OR, 1.27; 95% CI, 1.13– 1.43; *P*_FDR_ = .012 and G-CSF: OR, 1.70; 95% CI, 1.28–2.27; *P*_FDR_ = .027). The association between MICB_MICA and COPD risk remained in other MR approaches (Table S3, Supplemental Digital Content, https://links.lww.com/MD/O875). Besides, there was negative association of Septin-8, Corticosteroid-binding globulin (CBG), Butyrophilin subfamily 1 member A1 (BTN1A1) and Dystroglycan (DAG1) with overall COPD risk (Septin-8: OR, 0.68; 95% CI, 0.57–0.79; *P*_FDR_ = 8.00 × 10^−4^; CBG: OR, 0.81; 95% CI, 0.74–0.89; *P*_FDR_ = .005; BTN1A1: OR, 0.82; 95% CI, 0.75–0.90; *P*_FDR_ = .010 and DAG1: OR, 0.51; 95% CI, 0.35–0.73; *P*_FDR_ = .024). More details were presented in Tables S3, Supplemental Digital Content, https://links.lww.com/MD/O875, S5, Supplemental Digital Content, https://links.lww.com/MD/O876, and S6, Supplemental Digital Content, https://links.lww.com/MD/O877.

In terms of the association between proteins and COPD subtypes, we found BTN1A1, Tumor necrosis factor alpha-induced protein 8 (TNFAIP8), Dystroglycan (DAG1), and Switch-associated protein 70 would decrease the risk of early-onset COPD risk (Fig. 2B). There was a protective effect of BTN1A1 on early COPD incidence (OR, 0.72; 95% CI, 0.63–0.83; *P*_FDR_ = .002). Alpha-1-antitrypsin and Drebrin were negatively associated with the later-onset COPD risk, while C-X-C motif chemokine 17 showed a positive causal association (Fig. 2C and Tables S4, Supplemental Digital Content, https://links.lww.com/MD/O878; S5, Supplemental Digital Content, https://links.lww.com/MD/O876; S6, Supplemental Digital Content, https://links.lww.com/MD/O877).

### 3.2. Co-localization analysis

To validate the causal relationships between proteins and COPD and its subtypes, co-localization analysis was performed to detect the shared genetic variants in certain regions. The analysis found the PP.H4 of the causality of TIE-1 with COPD larger than .80, suggesting that there was a shared genetic variant and causal association. Additionally, BTN1A1 was colocalized with early-onset COPD with strong evidence (PP.H4 for BTN1A1 = .831). Meantime, the results of the colocalization suggested that there was a shared genetic variant of TNFAIP8 and DAG1 with early-onset COPD (all PP.H4 > .8). However, there was no evidence indicating a shared causal variant between the other proteins and COPD and its subtypes in these regions (all PP.H4 < .8, Table 1).

**Table 1:** Co-localization analysis for the causal association of protein and COPD.

Protein	Outcome	Number of SNP in the region	PP.H4.abf
TIE-1	COPD	6741	.865*
DAG1	COPD	4146	.742
CSF3	COPD	6592	.386
SEPTIN-8	COPD	7155	.253
MICB_MICA	COPD	18,968	.040
BTN1A1	COPD	7957	.007
SERPINA6	COPD	9314	9.40E−13
TNFAIP8	Early-onset COPD	9455	.858*
BTN1A1	Early-onset COPD	7957	.831*
DAG1	Early-onset COPD	4146	.804*
SWAP70	Early-onset COPD	8609	.660
DBN1	Later-onset COPD	6985	.770
CXCL17	Later-onset COPD	7628	.630
SERPINA1	Later-onset COPD	9370	.004

A higher PP.H4 supports significant MR results and are indicated in bold.

BTN1A1 = butyrophilin subfamily 1 member A1, COPD = chronic obstructive pulmonary disease, CSF3 = colony-stimulating factor3, DAG1 = dystroglycan, DBN1 = drebrin, MICB_MICA = MHC class I polypeptide-related sequence B_A, MR = Mendelian randomization, PP.H4 = posterior probability of the H4, SERPINA = Serpin A, SNP = single nucleotide polymorphisms, SWAP70 = switch-associated protein70, TIE-1 = tyrosine-protein kinase receptor Tie-1, TNFAIP8 = tumor necrosis factor alpha-induced protein 8.

*PP.H4 > .8 was defined as supporting evidence of co-localization analysis.

### 3.3. Phenome-wide MR analysis

**Figure 3.**
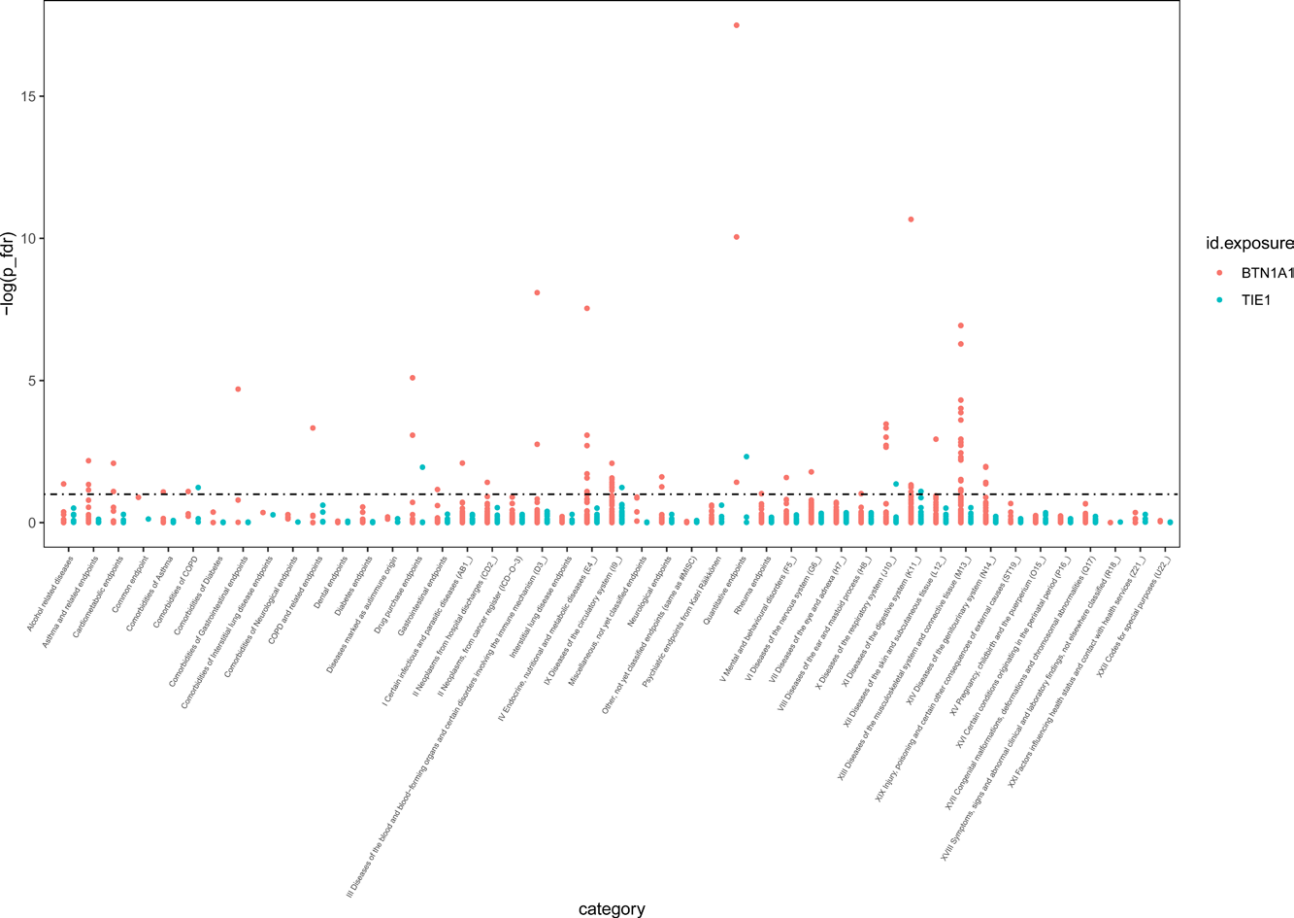
The effects of BTN1A1 and TIE-1 on phenotypes excluding overall COPD risk and its subtypes. BTN1A1 = butyrophilin subfamily 1 member A1, COPD = chronic obstructive pulmonary disease, TIE-1 = tyrosine-protein kinase receptor tie-1.

To comprehensively detect the side effect of the BTN1A1 and TIE-1, we further conducted the Phe-MR analysis. We estimated the effect of BTN1A1 and TIE-1 on 2403 phenotypes excluding overall COPD risk and its subtypes (Fig. 3). There were 58 clinic traits related to the BTN1A1, of which 6 phenotypes (schizophrenia, subacute thyroiditis, thyroiditis, and follicular lymphoma) showed positive association. BTN1A1 was inversely associated with the other phenotypes (Table S7, Supplemental Digital Content, https://links.lww.com/MD/O874). For instance, BTN1A1 would increase the subacute thyroiditis risk (OR, 3.28; 95% CI, 2.30–4.69; *P*_FDR_ = 2.89 × 10^−8^). Additionally, BTN1A1 was related to the increased risk of thyroiditis (OR, 1.93; 95% CI, 1.52–4.69; *P*_FDR_ = 2.01 × 10^−5^). Regarding to TIE-1, we only found 2 traits were related to it (Fig. 3), including height and antihypertensive medication.

## 4. Discussion

This is a large-scale study combining MR, colocalization, and Phe-MR analysis for the causal investigation of plasma proteins on COPD. We identify 7 COPD-associated proteins, of which 4 Septin − 8, CBG, BTN1A1, and DAG1 were negatively associated with COPD progression, whereas the remaining 3 (MICB_MICA, TIE-1, and G-CSF) were positively associated with COPD risk. Co-localization analysis further highlighted BTN1A1 and TIE-1 as potential drug targets for COPD, with their possible adverse effects evaluated through Phe-MR analysis.

BTN1A1 is part of B7-associated immunoglobulin family, which is crucial for immunomodulation,^[31]^ and is also involved in the secretion of milk fat droplets.^[32]^ It has also been shown to inhibit T-cell proliferation and reduce the levels of factors (e.g., IL-2 and IFNγ) that are associated with T-cell activation.^[33,34]^ In an animal experiment employing BTN1A1^-/-^ mice, deletion of BTN1A1 caused increased inflammation, and these highlight the role of BTN1A1 in immune regulation.^[35]^ In particular, BTN1A1 is also associated with xanthine dehydrogenase/oxidase,^[36]^ suggesting that it may be involved in a variety of cellular functions and biological pathways. There is limited existing research on the association between BTN1A1 with COPD. However, based on the fact that COPD is closely related to inflammation, BTN1A1 will have the potential to be a new target for COPD diagnosis and treatment. It has been shown that dendritic cells in the epithelial and submucosal layers of the trachea mucosa activate CD4^+^ and CD8^+^ T cells upon stimulation and are involved in the development of COPD.^[37,38]^ In a case-control study, an increased abundance of CD8^+^ T cells in the lungs of mild-to-moderate COPD associated with pre-severe lesions inflammation,^[39]^ aligning with our finding of a causal outcome of BTN1A1 with early COPD. IFN-γ stimulates the activation of alveolar macrophages and its level is positively associated with the severity of COPD.^[40,41]^ It has also been shown in a clinical study that patients with COPD combined with depression have the highest levels of blood IL-2 and IFN-γ compared to controls.^[42]^ Smoking and other factors are also predisposing factors for COPD. Smoke has been reported to upregulate the level of xanthine oxidase, which in turn causes lung vascular endothelial cell injury.^[43,44]^ Therefore, BTN1A1 may influence COPD progression via xanthine dehydrogenase/oxidase activity. These insights imply that BTN1A1 could be involved in multiple mechanisms of COPD, particularly in its early stages. This is clinically significant given the challenges of early COPD diagnosis.

TIE-1, a receptor tyrosine kinase on the cell membrane, consists of 4 functional domains. This protein is highly expressed in vascular endothelial cells. It plays a regulatory role in vascular development and neovascularization. It has also been reported to mediate tumor invasion and metastasis and to be involved in cisplatin resistance mechanisms.^[45,46]^ While no direct causal link between T1EI and COPD has been established, an indirect association can be inferred from the known pathogenesis of COPD and reported T1EI activity. Bronchial mucosal vascular proliferation is one of the characteristics of COPD. Vasodilation with capillary filling and leakage causes airway narrowing and promotes inflammatory cell infiltration, causing airflow obstruction.^[47]^ Inflammatory cells in COPD secrete large amounts of angiogenic factors, such as VEGF-A, VEGF-C, and VEGF-D. Structural cells also contribute to the pro-inflammatory and angiogenic effects in COPD.^[48]^ Thus, COPD appears to be a vascular disease as well. Smoke irritation affects the pulmonary vascular system and promotes vascular remodeling, which in turn causes pulmonary hypertension and induces complications such as pulmonary heart disease.^[49]^ Inhibition of bronchial vascular remodeling to control COPD may become a major direction of drug research. The revelation of the causal relationship between TIEI and COPD is new and never reported before, and more in-depth studies are needed to examine its feasibility as a therapeutic target.

MICB_MICA is expressed on malignant cells and its genetic variation is strongly associated with autoimmune diseases and susceptibility to infection.^[50,51]^ A significant increase in the proportion of NK cells in sputum and bronchial alveoli has been reported in COPD patients.^[52]^ After the organism is stimulated by infection, DNA damage or oxidative stress, MICA_MICB is expressed on the cells and binds to the receptor, causing NK cell activation and promoting COPD progression.^[53]^ Experimental study also showed that MICA expression was significantly upregulated in peripheral lung tissues of COPD patients, and MICA_MICB may be able to be used as a lung epithelial danger signal as an observable indicator for diagnosis and treatment.^[54]^ Septin-8 is a member of the Septin family of small GTPases that mediate processes such as cytokinesis and cytoskeletal remodeling.^[55]^ An epigenomic association study showed that hypomethylation of the SEPT8 gene was exhibited in patients with Asthma COPD overlap, suggesting that it has the potential to be a target for respiratory diseases.^[56]^ CBG is a carrier and reservoir for cortisol transport and plays an important role in metabolism, neuromodulation, and regulation of immune function.^[57]^ CBG targets cortisol to the site of inflammation to suppress inflammation and inhibit the process of organ damage, which is immunoprotective for the organism.^[58]^ Therefore, CBG may exert a protective effect on the organism by interfering with the inflammatory state of COPD, which is in line with the results that we obtained on the negative correlation between CBG and COPD. DAG1 is laminin-binding protein, consisting of 2 subunits, α-DG and β-DG.^[59]^ It plays a key role in skeletal muscle stabilization and also plays a unique role in processes such as cancer progression and cell signaling.^[59]^ An animal experiment showed that ROS-induced oxidative stress downregulated β-DG levels, which is beneficial for the progression of hypertension. Oxidative stress is also one of the pathogenic mechanisms of COPD, and DAG1 has the potential to serve as a target for therapy.^[60]^ G-CSF, a glycoprotein growth factor, is currently the most crucial drug in the clinical treatment of neutropenia.^[61]^ The primary inflammatory cells in the lungs of many COPD patients are neutrophils and serum G-CSF correlates with sputum IL-1β levels. G-CSF enrichment affects neutrophil function. A clinical trial has shown that high serum levels of G-CSF are strongly associated with acute exacerbations of COPD characterized by neutrophilic inflammation, and may serve as a diagnostic marker.^[62]^ Our results, along with those of existing studies, exemplify the complex relationship between these 5 proteins and COPD. However, extensive studies are still needed to elucidate heir precise roles in the disease and to establish a more dependable foundation for their use in diagnosis and therapeutic targeting.

This MR study combining pQTL and COPD GWAS data, provides novel and unique insights into identifying diagnostic markers and drug targets for COPD with the largest sample size pQTL data. This study combines multiple methodologies to minimize confounding and enhance reliability. First, MR analysis was used to reduce confounding and reverse causality bias. Meanwhile, cis-pQTL was analyzed as an instrumental variable, which provided higher confidence than trans-pQTLs and eQTLs. Second, co-localization analysis was performed to improve the confidence of the results. Finally, Phe-MR analysis was used to explore the side effects of potential drug targets in depth. However, some limitations of this study cannot be ignored: The data cohort analyzed included only European populations, limiting the generalizability of its application to the entire human population. Because the limited amount of COPD-related GWAS data does not allow for reproducibility, further studies are needed to validate the results obtained. There is variability in the populations included in the databases, which creates heterogeneity and may impact the results. Only the role of plasma proteins in COPD was assessed, but not the levels of related proteins in the lungs or other tissues. Although this study provides a novel and valuable reference for diagnostic markers and drug targets in COPD, additional experimental studies addressing these shortcomings are needed to deepen the knowledge of COPD disease progression and increase the likelihood of clinical translation of the results.

## 5. Conclusion

The study revealed the causal relationship between several plasma proteins and COPD, offering new theoretical support for understanding COPD. It also identified new markers and potential targets for the clinical diagnosis and treatment of COPD. Further studies evaluate the effectiveness of these candidate targets in subsequent studies to understand their potential in the clinical treatment of COPD.

## Acknowledgments

We extend our gratitude to the UK Biobank Pharma Proteomics Project and the Finnish database for providing the summary statistics essential for Mendelian randomization analyses. We also acknowledge the invaluable contributions of the researchers who shared these data, as well as all the authors who participated in this study.

## Author contributions

**Conceptualization:** Baicun Li.

**Data curation:** Xu Chu.

**Formal analysis:** Zhifa Han.

**Writing – original draft:** Xu Chu.

**Writing – review & editing:** Ting Yang.

## Supplementary Material


